# The MRZ-Reaction and Specific Autoantibody Detection for Differentiation of ANA-Positive Multiple Sclerosis From Rheumatic Diseases With Cerebral Involvement

**DOI:** 10.3389/fimmu.2019.00514

**Published:** 2019-03-19

**Authors:** Nils Venhoff, Jens Thiel, Marta Rizzi, Ana Venhoff, Sebastian Rauer, Dominique Endres, Carolin Hentze, Julian Staniek, Daniela Huzly, Reinhard E. Voll, Ulrich Salzer, Tilman Hottenrott

**Affiliations:** ^1^Department of Rheumatology and Clinical Immunology, Faculty of Medicine, Medical Center – University of Freiburg, Freiburg im Breisgau, Germany; ^2^Department of Neurology and Neurophysiology, Faculty of Medicine, Medical Center – University of Freiburg, Freiburg im Breisgau, Germany; ^3^Department of Psychiatry and Psychotherapy, Faculty of Medicine, Medical Center – University of Freiburg, Freiburg im Breisgau, Germany; ^4^Faculty of Medicine, Institute of Virology, Medical Center – University of Freiburg, Freiburg im Breisgau, Germany

**Keywords:** anti-nuclear antibodies, systemic lupus erythematosus (SLE), multiple sclerosis (MS), intrathecal polyspecific antiviral immune response, MRZ-reaction (MRZR)

## Abstract

**Objective:** Rheumatic diseases with involvement of the central nervous system (RDwCNS) may mimic multiple sclerosis (MS). Inversely, up to 60% of MS-patients have antinuclear autoantibodies (ANAs) and may be misdiagnosed as RDwCNS. The detection of antibodies against extractable nuclear antigens (ENA) and oligoclonal bands (OCB) are established valuable diagnostic tools in the differential diagnosis of RDwCNS and MS. The MRZ-reaction (MRZR) is defined by three antibody indices (AIs) against neurotropic viruses and is frequently positive in MS. To investigate the added value of MRZR combined with testing for antibodies against ENAs and OCB detection to distinguish RDwCNS from ANA positive MS.

**Methods:** MRZR was evaluated in RDwCNS (*n* = 40) and 68 ANA positive MS-patients. Two stringency levels, MRZR-1 and MRZR-2 (at least one respectively two of three AIs positive) were applied. Autoantibody testing included ANA plus ENA and anti-dsDNA antibodies, antiphospholipid antibodies, and anti-neutrophil cytoplasmic antibodies.

**Results:** Most of the RDwCNS patients (*n* = 32; 80%) suffered from systemic lupus erythematosus. Within the RDwCNS group 20% had a positive MRZR-1 and 8.5% a positive MRZR-2 compared to 80.9 and 60%, respectively within the MS-group (*p* < 0.0001 for both comparisons). Oligoclonal bands were found in 28.6% of the RDwCNS patients and 94.3% of the MS-patients (*p* < 0.0001). Conversely, autoantibodies to specific nuclear antigens or phospholipids were found more frequently in RDwCNS. A positive MRZR in conjunction with the absence of ENA autoantibodies distinguished MS from RDwCNS with high specificity (97.5%).

**Conclusions:** We suggest combining MRZR, OCBs, and specific autoantibody diagnostics to differentiate RDwCNS from MS.

## Introduction

Multiple sclerosis (MS) is an immune-mediated inflammatory disease of the central nervous system (CNS). MS and rheumatic diseases show several commonalities. Between 20 and 60% of MS-patients have a positive immunofluorescence testing for anti-nuclear antibodies (ANAs) (1, 2) and in rheumatic diseases such as systemic lupus erythematosus (SLE) or anti-neutrophil cytoplasmic-antibodies (ANCA) associated small vessel vasculitides, neuropsychiatric manifestations are common and may mimic magnetic resonance imaging (MRI), and cerebrospinal fluid (CSF) findings of MS (3, 4). As neuropsychiatric symptoms may be the first clinical manifestation of rheumatic diseases with involvement of the central nervous system (RDwCNS) and inflammatory CSF alterations and MS-like lesions in MRI are frequent, the differential diagnosis between RDwCNS and ANA-positive MS is difficult. Moreover, differentiating RDwCNS from ANA-positive MS is essential for adequate treatment. Also the coexistence of both a systemic inflammatory rheumatic disease and MS in the same patient has to be considered since both entities are of autoimmune origin and occur predominantly in female patients. One essential diagnostic procedure in these patients is the analysis of CSF which requires lumbar puncture. The MRZ-reaction (MRZR) is a polyspecific, intrathecal humoral immune response directed against three neurotropic viruses: measles (M), rubella (R), and varicella zoster (Z), assessed using the three respective antibody indices (AIs) (5). The AI is a calculated parameter to assess whether the antibodies measured in the CSF are produced intrathecally or whether they are originally blood derived. A high AI (≥1.5) is an indicator for antibody production within the CNS whereas an AI < 1.5 is indicative for an antibody synthesis in plasma cells that are not located within the CNS. In MS studies frequently two thresholds defining a positive AI (≥1.5 and >2.0) are assessed (6). Furthermore, it is common to distinguish a positive MRZR-1 and a positive MRZR-2. A positive MRZR-1 is defined by at least one positive AI and a positive MRZR-2 by at least two positive AIs out of the three calculated AIs. Very likely the positive MRZR represents a polyspecific B-cell-activation within the CNS. Also the detection of oligoclonal bands (OCB), a very sensitive but compared to the MRZR less specific marker for MS, is an indicator for the involvement B-cells in the pathogenesis of MS.

A high prevalence of positive MRZR has been described in patients with relapsing remitting MS and with primary progressive MS (6), while the significance of positive MRZR in RDwCNS has not yet been explored in larger cohorts. Since ANA can be detected also in healthy individuals, a positive ANA-screening should lead to an analysis of extractable nuclear antigens (ENA). Certain antibodies to ENA are highly specific for connective tissue diseases (CTD), whereas the absence of ENA or exclusive detection of DFS70-autoantibodies in ANA-positive individuals does not further support the diagnosis of an underlying CTD (7). Therefore, the MRZR together with ANA and ENA testing might represent a valuable diagnostic procedure to separate MS from RDwCNS. This is the first report on the diagnostic value of the MRZR in combination with ENA-autoantibody diagnostics to differentiate RDwCNS-patients from ANA-positive MS in the largest cohort of RDwCNS-patients published so far.

## Materials and Methods

Patients participating in this retrospective study were treated at the University Medical Centre Freiburg and were identified by an electronic database search. Routine medical diagnostic workup included lumbar puncture in all patients and the storage (−80°C) of paired CSF and serum samples according to local biobanking protocols. Informed consent was obtained from all patients. All experiments were carried out in accordance with the Declaration of Helsinki. This study was approved by the ethics committee of the University Medical Centre Freiburg (EK-Fr489/14, EK-Fr507/16). Diagnoses of the rheumatic diseases were made by board certified rheumatologists according to current classification criteria (8–11). CNS-involvement of RDwCNS was diagnosed based on clinical signs, and the presence of at least one of the following findings: (A) inflammatory CSF (intrathecal immunoglobulin synthesis, increased cell-count, positive CSF specific oligoclonal bands (OCB), or disturbance in the blood-CSF barrier indicated by an increased albumin quotient) or (B) inflammation in brain or spinal MRI compatible with RDwCNS as assessed by board-certified neuroradiologists. MS-diagnosis was made according to the 2010 revised McDonald criteria (12). Total immunoglobulin concentrations (IgG_total_) were measured by nephelometry (BN-ProSpec System, Siemens, Germany). Measles-, rubella-, and varicella zoster-specific IgG concentrations (IgGspec) were measured using ELISA (Serion *classic* ELISA, Germany). MRZR was calculated from the virus-specific antibody index (AI) = QIgG_spec_/QIgG_total_, if QIgG[total] < Qlim, and AI = QIgG[spec]/Qlim, if QIgG[total]>Qlim (13). The upper reference range of QIgG, Qlim, was calculated according to Reiber's formula (13). Two thresholds for a positive AI indicating specific intrathecal IgG-production (≥1.5 and >2.0) were analyzed (6, 14). MRZR-1 and MRZR-2 were positive when at least one respectively two of the three calculated AIs were positive. ANA-staining pattern was assessed using indirect immunofluorescence (IIF) on HEp-2000® cells (Immuno Concepts, Sacramento, CA, USA). Patients with positive IIF were screened for autoantibodies against ENA using a lineblot assay including nRNP/Sm, Sm, SS-A, Ro-52, SS-B, Scl-70, PM-Scl,Jo-1, CENP-B, PCNA, dsDNA, nucleosomes, histones, ribosomal-P-proteins, AMA-M2, and DFS70 (ANA-Profile3plusDFS70, Euroimmun, Luebeck, Germany) and an anti-dsDNA-IgG-ELISA (Euro-Diagnostica, Malmö, Sweden). Anti-phospholipid-antibodies (Cardiolipin-IgG-ELISA, Euro-Diagnostica, Malmö, Sweden) and anti-proteinase-3 (Orgentec Diagnostika, Mainz, Germany) or myeloperoxidase (Euroimmun, Luebeck, Germany) was measured using ELISA. Two or more OCB detected by an isoelectric focusing technique (Hydragel Isofocusing, Sebia, France) were regarded as positive (15). Statistical analyses were performed using Fisher's exact test (two-sided) and Student's *t*-test (two-sided) with a *p* < 0.05 regarded as statistically significant (Graphpad Prism version 7.01).

## Results

ANAs were assessed by IIF in a cohort of 149 MS-patients. We found 68 MS-patients (45.6%) with positive ANA and we compared them with 40 RDwCNS-patients. The RDwCNS-group consisted of 32 patients with SLE (80%), six with ANCA-associated vasculitis (15%), one with Cogan's syndrome and one with Behcet's disease. All RDwCNS patients fulfilled the classification criteria for their underlying rheumatic disease and showed signs of CNS involvement (definition see above). Except for the patients with Cogan's syndrome, Behcet's disease and one patient with ANCA-associated vasculitis all had at least one specific autoantibody supporting the diagnosis of the rheumatic disease. The diagnosis was also supported by concomitant non-neurological manifestations at the skin/mucosa (*n* = 34), joints (*n* = 26), blood/cytopenia (*n* = 15), peripheral nervous system (*n* = 7), ear-nose-throat-involvement (*n* = 5), pericarditis/pleuritis (*n* = 5), or lung-involvement (*n* = 4). Both groups were similar regarding age and sex (Table 1). In both, the MS- and the RDwCNS-group, < 40% of patients had total CSF cell-counts above 5/μl. The RDwCNS-group though showed higher mean CSF cell-count (cells/μl) and higher frequency of patients with high CSF cell counts (>50 cells/μl) (*p* < 0.05 for both comparisons). The proportion of patients with increased intrathecal immunoglobulin synthesis (IgG, IgM, or IgA) and positive OCBs, both used as diagnostic parameters for MS, was significantly higher in MS-patients (*p* < 0.001 for both comparisons). Within the MS-group positive OCBs were found in 89.7% which was more frequent than a positive MRZR-1 (80.1%). Nevertheless positive OCBs and intrathecal immunoglobulin synthesis were also found in approximately 30% of our RDwCNS-patients. With respect to serological findings ANA-positive MS-patients had a lower median ANA serum titer compared to RDwCNS. Correspondingly, specific autoantibodies directed against nuclear antigens (ENA-analysis) were more frequent in RDwCNS (see Table 1).

Table 1Patient characteristics, serological findings, and MRZ-reaction.

**Table:** 

	**RDwCNS (*n* = 40)**	**MS (*n* = 68)**	**Statistics (*p*-value)**
Gender, female, *n* (%)	30 (75)	48 (71)	n.s.
Mean age years (range, SD)	45.7 (19–79, 19.1)	44.9 (23–73, 12.3)	n.s.
**CEREBROSPINAL FLUID ANALYSIS RESULTS**			
Increased total CSF cell count (>5/μl), *n* (%)	15 (37.5)	20 (29.4)	n.s.
Mean cell count/μl in CSF, (range, SD)	31 (1–433, 84)	6 (1–44, 8)	*p* = 0.0176
Cell count >50/μl, *n* (%)	4 (10)	0 (0)	*p* = 0.0171
Intrathecal synthesis of IgG, IgM, or IgA, *n* (%)	11 (27.5)	43 (63.2)	*p* = 0.0006
Oligoclonal bands, *n* (%)	13 (32.5)	61 (89.7)	*p* < 0.0001
**SEROLOGICAL FINDINGS**			
Autoantibody positive, *n* (%)	38 (95)	68 (100)	n.s.
IIF ANA positive, *n* (%)	33 (82.5)	68 (100)	n.s.
Median ANA titer (IQR, range)	800 (400–3200; 200–6400)	400 (200–700; 100–3200)	*p* = 0.0035
Anti-dsDNA, *n* (%)	22 (55)	3 (3.8)	*p* < 0.0001
Anti-nucleosome/anti-PCNA-antibodies, *n* (%)	13 (32.5)	0 (0)	*p* < 0.0001
Anti-SS-A/Ro, SS-B/La-antibodies, *n* (%)	6 (15)	0 (0)	*p* < 0.0001
Anti-centromere, anti-Scl70 antibodies, *n* (%)	3 (7.5)	0 (0)	*p* = 0.0352
DFS70-antibodies, *n* (%)	0 (0)	2 (2.9)	n.s.
ANCA, *n* (%)	5 (12.5)	1 (1.5)	*p* = 0.0254
APA, *n* (%)	12 (30)	2 (2.9)	*p* < 0.0001
**MEASLES-RUBELLA-ZOSTER-(MRZ)-REACTION**			
Mean AI for M (range, SD)	1.2 (0.6–4.9; 0.7)	3.1 (0.5–22.7; 3.3)	*p* = 0.0007
Mean AI for R (range, SD)	1.2 (0.6–5.4; 0.8)	3.3 (0.5–22.7; 4.1)	*p* = 0.0021
Mean AI for Z (range, SD)	1.3 (0.6–4.2; 0.8)	2.3 (0.7–11.9; 2.3)	*p* = 0.008

**Table:** 

**FREQUENCY OF POSITIVE ANTIBODY INDECES (AIs) FOR MEASLES, RUBELLA, ZOSTER**						
**Applied threshold defining a positive AI**	**≥1.5**	**>2.0**	**≥1.5**	**>2.0**	**≥1.5**	**>2.0**
Positive AIs (**M**easles), *n* (%)	4 (10)	2 (5)	40 (58.8)	31 (45.6)	*p* < 0.0001	*p* < 0.0001
Positive AIs (**R**ubella), *n* (%)	4 (10)	2 (5)	33 (48.5)	27 (39.7)	*p* < 0.0001	*p* < 0.0001
Positive AIs (**Z**oster), *n* (%)	5 (12.5)	3 (7.5)	30 (44.1)	21 (30.9)	*p* = 0.0006	*p* = 0.0043
**FREQUENCY OF PATIENTS WITH 0, 1, 2, 3 POSITIVE ANTIBODY INDECES (AI)**						
**Applied threshold defining a positive AI**	**≥1.5**	**>2.0**	**≥1.5**	**>2.0**	**≥1.5**	**>2.0**
0 positive AI, *n* (%)	32 (80)	35 (88)	13 (19.1)	21 (30.9)	*p* < 0.0001	*p* < 0.0001
1 positive AI, *n* (%)	3 (7.5)	3 (7.5)	18 (26.5)	22 (32.4)	*p* = 0.0221	*p* = 0.0039
2 positive AIs, *n* (%)	3 (7.5)	1 (2.5)	19 (27.9)	16 (23.5)	*p* = 0.0128	*p* = 0.0048
3 positive AIs, *n* (%)	2 (5)	1 (2.5)	18 (26.5)	9 (13.2)	*p* = 0.0049	n.s.

*AI, antibody index; ANA, antinuclear antibody; ANCA, anti-neutrophil cytoplasmic antibodies; APA, antiphospholipid antibodies; dsDNA, double stranded DNA; IIF, indirect immunofluorescence; M, measles; MS, multiple sclerosis; n, number of patients; n.s., not significant; RDwCNS, rheumatic diseases with involvement of the central nervous system; R, rubella; SD, standard deviation; Z, varicella zoster. The bold values are the applied thresholds (>1.5 or >2.0) defining a positive AI for the calculation of the MRZR*.

**Figure 1:**
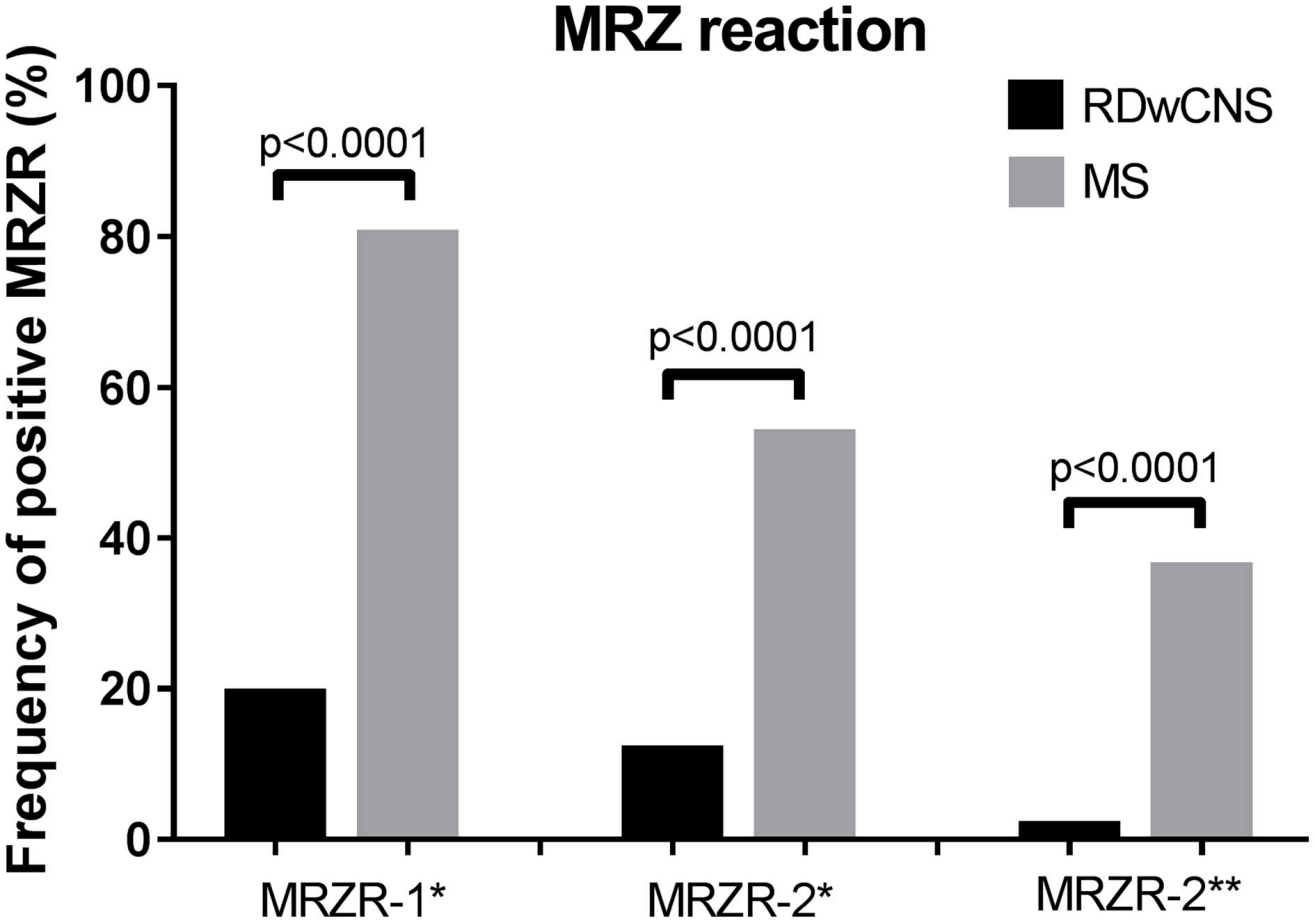
MRZ reaction in RDwCNS patients compared to MS patients. Illustrated is the frequency of a positive MRZR-1 and MRZR-2 calculated with an AI defined positive when ≥1.5 (^*^) or >2.0 (^**^). MRZR, MRZ reaction; MS, multiple sclerosis; RDwCNS, rheumatic disease with CNS involvement.

Mean AIs for measles, rubella, and zoster were significantly higher in MS-patients compared to RDwCNS-patients (Table 1). Positive AIs, irrespectively of the thresholds used for definition (>1.5 and >2.0) were found with a higher frequency in ANA-positive MS compared to RDwCNS for all three specificities. Within the MS-group AIs were most frequently positive for measles, followed by rubella and varicella zoster. This AI-distribution pattern was not found within the RDwCNS-group. The MRZR-1 (AI positive when ≥1.5) was positive in 80.9% of ANA-positive MS-patients and 20% of RDwCNS-patients (*p* < 0.0001), the MRZR-2 was positive in 54.4% compared to 12.5% in RDwCNS (*p* < 0.0001) (Figure 1). By using the higher threshold of >2.0 for a positive AI, the prevalence of a positive MRZR-2 dropped to 2.5% (*n* = 1) within the RDwCNS-group compared to 36.8% within the MS-group (*p* < 0.0001).

Within the ANA-positive MS-group 55 of the 68 patients (80.9%) had a positive MRZR-1 but only seven MS-patients (10.3%) had specific autoantibodies. When combining both biomarkers, a positive MRZR-1 and the absence of autoantibodies against specific autoantigens, statistical analysis showed an increased specificity of 97.5% and an only slightly decreased sensitivity of 75% for the diagnosis of MS.

## Discussion

Rheumatic diseases with involvement of the central nervous system are a diagnostic challenge, especially if CNS-involvement is the first or only manifestation. Furthermore, RDwCNS has to be distinguished from rheumatic diseases with co-manifestation of MS. Not only clinically, but also by using imaging diagnostics and CSF analyses it is often difficult to distinguish RDwCNS clearly from MS, especially within the early disease course of MS and when autoantibodies like ANA are present in MS-patients (1–3).

Antinuclear antibodies, which are the hallmark of connective tissue diseases, were found in nearly half of our MS-cohort, a frequency within the published range between 20 and 60% (1, 2). Also ANCA, crucial in the diagnostics of ANCA-associated vasculitides were found to be present in a small proportion of MS-patients. Since testing for specific autoantibodies (extractable nuclear antigens, dsDNA, anti-phospholipid-antibodies, anti-proteinase 3- or anti-myeloperoxidase-antibodies) was positive in only 10% of the ANA-positive MS-patients, ANA-diagnostics should always comprise both, indirect immunofluorescence for screening and immunoblot/ELISA for differentiation of antibodies against nuclear antigens, to distinguish RDwCNS from ANA-positive MS. ANA-differentiation should include DFS70-antibodies. If DFS70-antibodies are detected exclusively in an ANA-positive patient, the positive ANA-test does not increase the likelihood for a CTD (7). We found statistically significant differences between RDwCNS and MS regarding CSF cell count, the presence of OCBs and the production of intrathecal immunoglobulins, but none of these parameters was able to reliably differentiate ANA-positive MS from RDwCNS, when used exclusively. It has already been shown that a positive MRZR has a higher specificity than the presence of OCBs for the diagnosis of MS, while positive OCBs have a high sensitivity but quite low specificity. In line with these data we found OCBs more frequent than a positive MRZR-1 in our MS-group but also in a relatively high frequency in our RDwCNS-cohort. Even when combining MRI, CSF and clinical findings it can be difficult to differentiate MS from RDwCNS. Therefore, only the combination of several diagnostic parameters established for MS and for RDwCNS, may result in a diagnostic algorithm with sufficient sensitivity and specificity to distinguish between both disease entities. MRZR is already established as a valuable diagnostic tool in MS, but to date it is not used to differentiate RDwCNS from ANA-positive MS. Therefore, we aimed to include MRZR in the diagnostic algorithm in addition to already established diagnostic procedures (e.g., OCBs). MRZR was found positive more frequently in MS than in RDwCNS, despite a high frequency of autoantibodies, hypergammaglobulinemia and positive OCBs in RDwCNS. This is in accordance with findings described before in MS-patients independently of their ANA-status (14, 16, 17). Especially in combination with the absence of specific autoantibodies to nuclear antigens or lack of ANCA-reactivity against PR3 or MPO, MRZR-1 yielded a high specificity and a good sensitivity for diagnosis of MS. Since DFS70-autoantibodies, which help to exclude CTDs, were positive in only 2.5% of the MS-group, this test was of no further diagnostic value in ANA-positive MS-patients (7).

Three female patients in our RDwCNS-group had a positive MRZR-2 reaction (threshold ≥1.5). In one of these the MRZR remained positive even when a threshold >2.0 was applied, making it difficult to exclude the coexistence of both SLE and MS. Unfortunately, CSF diagnostics, electrophysiological tests, MRI, and the pattern of non-neurological clinical manifestations were not sufficient to exclude MS in this patient. In conclusion, we found a positive MRZR in a large proportion of ANA-positive MS-patients but in very few RDwCNS-patients. MRZR seems to be less sensitive but more specific than OCBs for the diagnosis of MS. Especially, when specific autoantibodies are absent, a positive MRZR yields a high specificity with good sensitivity. Therefore, we recommend including both, the MRZR and autoantibody screening for ENA, as parameters additionally to the established parameters like OCB in the diagnostic algorithm for differentiation of RDwCNS from ANA-positive MS.

## Data Availability

All datasets generated for this study are included in the manuscript and/or the supplementary files.

## Author Contributions

NV, JT, US, DE, and TH designed and initiated this study. TH and SR identified the enrolled MS patients. NV, JT, CH, and RV identified the RDwCNS patients enrolled in this study. NV, TH, US, and JT performed the statistical analysis and drafted the manuscript. RV, MR, JS, DE, AV, and US helped in interpretation of the data and in drafting the manuscript. US and AV supervised the measurements of the rheumatic antibodies. DH supervised the performance of the immunoassays in the Institute of Virology. All authors have read the manuscript, contributed to manuscript revision, and approved the submitted version.

### Conflict of Interest Statement

The authors declare that the research was conducted in the absence of any commercial or financial relationships that could be construed as a potential conflict of interest.
